# A Cytological Study Enlightening the Unseen Effects of Concomitant Chemoradiotherapy in Contralateral Normal Buccal Mucosa of Oral Squamous Cell Carcinoma Patients

**DOI:** 10.7759/cureus.14483

**Published:** 2021-04-14

**Authors:** Sadia Minhas, Aneequa Sajjad, Maria Noor, Fariha Qureshi, Romaisa A Khokhar, Muhammad Kashif

**Affiliations:** 1 Microbiology, The University of Lahore, Lahore, PAK; 2 Oral Pathology, Akhter Saeed Medical and Dental College, Lahore, PAK; 3 Department of Oral Medicine, Fatima Memorial Hospital College of Medicine and Dentistry, Lahore, PAK; 4 Anatomy, Akhtar Saeed Medical and Dental College, Lahore, PAK; 5 Oral Pathology, Shifa College of Dentistry, Shifa Tameer e Millat University, Islamabad, PAK; 6 Oral Pathology, Bakhtawar Amin Medical and Dental College, Multan, PAK

**Keywords:** oral squamous cell carcinoma, concomitant chemo-radiotherapy, field cancerization, oral exfoliative cytology

## Abstract

Background/objectives

In patients receiving concomitant chemoradiotherapy (CCRT) as a treatment for oral squamous cell carcinoma (OSCC), cytological changes were seen not only in neoplastic epithelial cells but the non-neoplastic epithelial cells are also affected, resulting in cytopathological atypical changes. The present study was designed to observe oral epithelial atypical cytopathologic changes induced in contralateral normal buccal mucosa in OSCC patients receiving CCRT.

Methods

The study included 150 patients with OSCC treated by CCRT whose details were collected from the Institute of Nuclear Medicine and Oncology Lahore (INMOL) Hospital Lahore. Cytological smears were obtained from the contralateral normal buccal mucosa of OSCC patients. The serial scrape smears were taken before, immediately after, on the 17th day (mid of treatment), and at the end of CCRT, whereas 20 patients were taken as normal healthy controls and were not exposed to CCRT. The smears were stained with hematoxylin and eosin and Papanicolaou stain. SPSS version 20 (Armonk, NY: IBM Corp.) was used for statistical analysis and p > 0.05 was considered to be significant.

Results

CCRT-induced oral epithelial atypical cytological changes were predominantly noted at end of therapy (19.7%) in the contralateral normal buccal mucosa. Nuclear atypia features were higher on the 17th day and end of treatment; whereas, epithelial atypia was mainly observed on the 17th day of CCRT (40%). A highly significant association was observed between epithelial atypia and radio-chemotherapy dose (p = 0.045), between CCRT-induced epithelial atypical cytological changes and days of treatment (p = 0.001), and between days of CCRT and nuclear atypia (0.000) accordingly. Atypia was not observed in any control group.

Conclusion

Varying degrees of oral epithelial atypical cytological changes may occur in otherwise normal contralateral mucosa of the patients receiving CCRT.

## Introduction

Squamous cells that line the mucosal surfaces within the head and neck region usually give rise to the cancers of this region upon molecular and genetic insults [1]. Head and neck cancers (HNC) are additionally characterized according to the area of the head and neck from where they arise, and these consist of the pharynx, nasal cavity, larynx, oral cavity, salivary glands, and paranasal sinuses [1].

Globally, oral cancer (OC) is the sixth most common cause of cancer-related deaths, although many people are unaware of its presence [2]. Of these OCs, more than 90% are oral squamous cell carcinomas (OSCC) arising in the mucous membranes of the oral cavity and oropharynx [3].

Oral cancer is the second most common cancer after a bronchogenic tumor in males and breast tumor in females in Pakistan with an increase of about 16,000 new cases annually [4]. The 2018 Shaukat Khanum cancer registry reported OC in the top ten malignancies among all age groups of both genders [5]. According to findings from different research centers, the OC is one of the most common cancers in the province of Punjab, Pakistan with 714 cases of HNC were seen, and the foremost cancer was oral cavity 228 [6].

In the early stages, OC is usually treated with surgery and radiation therapy, whereas patients with advanced oral tumors may undergo a combination of treatments, i.e., concomitant chemoradiotherapy (CCRT). The OC patients may receive radiation therapy with surgery, which is administrated before, during, or after surgery. A combination of different chemotherapy drugs is used for the treatment of cancer. There is more chance of killing more cells with the use of more than one type of drug. The selection of treatments depends on patient general health, cosmetic and functional outcomes, and availability of expertise and resources [7].

CCRT regimen represents the most excellent current standard therapy for many patients with regionally advanced solid tumors, that improve the likelihood of cure. The clinical goal of administrating chemotherapy and radiation concurrently is to develop both locoregional, systemic tumor control with organ protection as well as to increase the high survival rates and to increase the efficacy of radiation, i.e., radiation sensitization [8].

In 1953, the field of cancerization was described as tissue adjacent to OSCC having abnormal histological tissue predominantly in the upper oropharyngeal tract most likely associated with exposure to different carcinogens. The advancement of numerous cancers is frequently linked with the thought of field cancerization in which multiple tumors appear at various distant locations because of gene alteration caused by carcinogens but not by metastasis [9].

The state of the epithelium changes after sustained contact with the carcinogens, leading to the development of carcinoma involving multiple areas, which can also originate without the involvement of genetic influence. This progression might enlighten the fact that there is an increased incidence of recurrence of carcinomas despite surgical and radiotherapy treatment. Because of alteration in the preconditioned epithelium, tumor recurrence is most frequently seen and is more susceptible to cancer lying adjacent to the site of a tumor eradicated by radiation therapy [10].

The oral cavity was recognized to be most prone to the process of field cancerization as the oral cavity comes in contact with a variety of environmental carcinogens which in turn disturb the oral mucosa leading to the occurrence of premalignant conditions at the same time [11]. The present study was conducted to define the atypical epithelial changes, their incidence, and type of field change in contralateral normal buccal mucosa with unilateral OSCC on different days of CCRT.

## Materials and methods

The study included 150 patients of OSCC treated by CCRT, collected from the Institute of Nuclear Medicine and Oncology Lahore (INMOL) hospital, Lahore according to inclusion and exclusion criteria. Patients of both genders of age 18 years and above receiving CCRT for OC for the first time were included in the study whereas patients with associated chronic ailments like tuberculosis, diabetes mellitus, immune disorders, patients with bilateral oral malignancies, or those who already underwent CCRT for already diagnosed OSCC were excluded from the study. The study was approved by the Advanced Studies and Research Board of the University of Health Sciences, Lahore. The samples were taken after taking Informed consent from the patients. Socio-demographic information along with relevant clinical details was recorded on specially designed proforma.

After detailed examination, cytological smears were obtained from the contralateral normal buccal mucosa of OSCC patients (also referred to as mirror image site in this study). The serial scrape smears were taken before CCRT, immediately after CCRT, on the 17th day of CCRT (mid of treatment), and at the end of CCRT presenting a distinctive view to study the CCRT response on the contralateral normal buccal mucosa, whereas 20 patients were taken as normal healthy control who were not receiving CCRT. The smears were then stained with hematoxylin and eosin and Papanicolaou stain. The smear adequacy is based on the presence of sufficient numbers of epithelial cells to provide reliable assessment [12] and the smears were studied by at least three histopathologists.

Epithelial atypia changes in the study population of the current research were evaluated cytologically by means of the criteria defined by Ahmed et al. [13]. The presence of two or more of the following characteristics was consistent with epithelial atypia: binucleation or multinucleation, pleomorphism of nucleus and cell, chromatin clumping with relatively prominent nucleoli, increase in the size of nucleus associated with raised nuclear-cytoplasmic (N/C) ratio. In this study, the term atypical epithelial change was used instead of mild dysplasia and were assessed by using the criteria mentioned by Babshet et al. and was concluded when two to three following features was observed: irregular nuclear membrane, increased N/C ratio, hyperchromasia, atypical mitotic figures, anisocytosis, and poikilocytosis [14].

The data were entered and analyzed using SPSS 20 (Armonk, NY: IBM Corp.). Mean + SD was given for quantitative variables like age, radiotherapy dosage, and chemotherapy dosage. Frequencies, percentages, and graphs were given for qualitative variables like pathological changes and nuclear changes. The data were analyzed by applying the Chi-square test and Fisher’s exact test. A p-value of <0.05 considered to be statistically significant.

## Results

**Figure 1:**
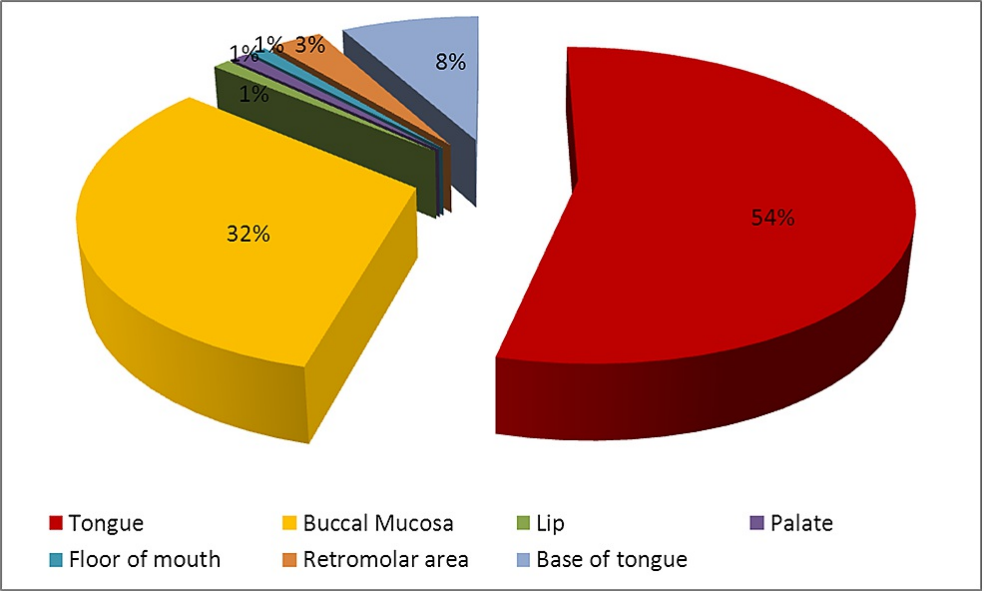
Site of involvement of oral cancer

The mainstream of patients was male n = 98 (65.3%) with male to female ratio of 1.8:1 with an overall mean age of the patients was 50.50 ± 11.3 years whereas most of the patients presented between the age ranges of 46-55 years with S.D ± 11.35. The predominant site of involvement was the tongue (53.9%) with the lateral border involved mostly, followed by buccal mucosa (31.6%; Figure 1).

When the OSCC were subclassified on the basis of their histological subtypes, 98.7% were conventional SCC while verrucous carcinoma was seen only in n = 2 (1.3%) cases. The descending order of histological grading of OSCC was moderately differentiated squamous cell carcinoma followed by well-differentiated squamous cell carcinoma and poorly differentiated squamous cell carcinoma in 53.9%, 30.3%, and 15.8% of cases accordingly.

Considering the fractions of radiotherapy dosages, patients received 70 Gy, 90 Gy, and 119 Gy. Most of the patients (51.3%) received a 90 Gy dose of radiotherapy. While considering the chemotherapy most of the patients n = 124 (82.6%) received combination drug therapy (Cisplatin and 5-Fluorouracil) whereas only 17.4% of patients received cisplatin for treatment.

The nuclear atypia features include karyolysis, karyorrhexis, nuclear budding, multinucleation, micronucleated, prominent nucleoli, binucleation, and pleomorphism. All these variables of nuclear atypia were predominantly observed in the study group at the 17th day and end of CCRT on contralateral normal buccal mucosa. The nuclear atypical changes were greater in the study group in comparison to the control group (Table 1).

**Table 1:** Distribution of nuclear atypia from contralateral buccal mucosa on a different day of concomitant chemoradiotherapy in the study and control group

Characteristics	Before the start of treatment	Immediate after treatment	On the 17th day of treatment	At the end of treatment	Control group
Pleomorphism	16 (10.6%)	62 (41.3%)	150 (100%)	150 (100%)	2(10%)
Karyolysis	2 (1.3%)	6 (4%)	150 (100%)	150 (100%)	1(5%)
Karyorrhexis	2 (1.3%)	4 (2.6%)	148 (98.6%)	150 (100%)	1(5%)
Binucleation	14 (9.3%)	64 (42.6%)	148 (98.6%)	150 (100%)	0
Micronucleation	6 (4%)	36 (24%)	150 (100%)	150 (100%)	1(5%)
Prominent nucleoli	30 (20%)	116 (77.3%)	150 (100%)	150 (100%)	2(10%)
Nuclear budding	0	0	32 (21.3%)	146 (97.3%)	0
Multinucleation	0	0	22 (14.6%)	116 (77.3%)	0

**Figure 2:**
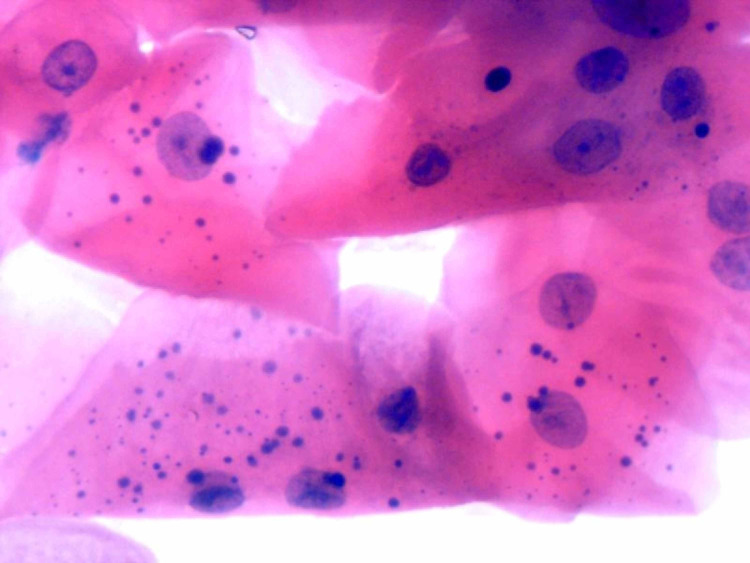
Photomicrograph from contralateral normal buccal mucosa exfoliated epithelial cells at the end of CCRT from OSCC patient showing features of therapy-induced epithelial atypia, i.e., prominent nucleoli, mild of hyperchromasia, micronuclei, and mild to moderate degree of hyperchromasia (H&E × 400) CCRT: concomitant chemoradiotherapy, OSCC: oral squamous cell carcinomas.

A total of 600 smears were taken from contralateral buccal mucosa at all four particular days of CCRT; it was noticed that epithelial atypia was present in n = 90 (15%) smears, whereas it was absent in the remaining smears in the study group. When we compare epithelial atypia on contralateral buccal mucosa among days of therapy, it was predominantly seen on the 17th day of treatment in n = 36 (24%) followed by an immediate day of therapy in n = 24 (16 %), end of CCRT n = 18 (12%), and before the start of CCRT n = 12 (8%) smears, whereas no evidence of epithelial atypia was detected among the control group (Figure 2).

**Figure 3:**
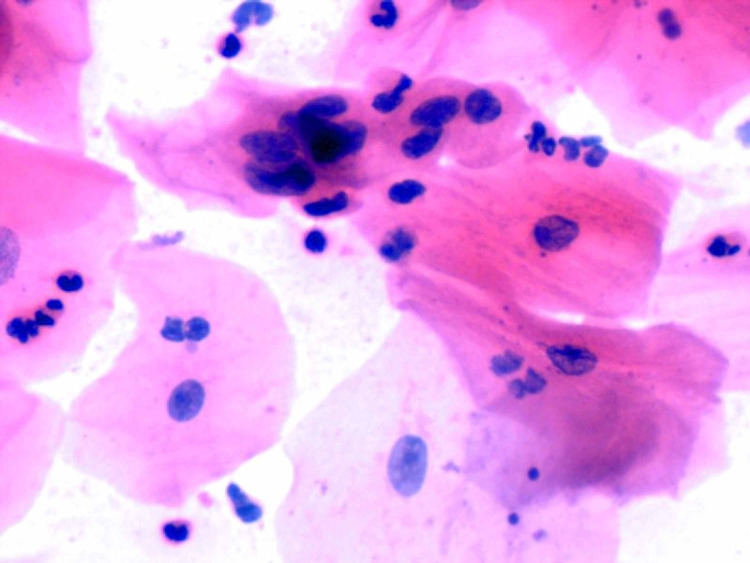
Showing mild to moderate degree of hyperchromasia, nuclear pleomorphism, high N/C ratio with irregular nuclear membranes, prominent nucleoli, and atypical mitosis suggesting CCRT-induced atypical epithelial changes in smear obtained from contralateral normal buccal mucosa at the end of CCRT from OSCC patient (H&E × 400) CCRT: concomitant chemoradiotherapy, OSCC: oral squamous cell carcinomas, N/C: nuclear-cytoplasmic, H&E: hematoxylin and eosin.

Among 600 smears from contralateral buccal mucosa on all particular days of CCRT, epithelial atypical changes were seen in n = 50 (8.3%) smears whereas in n = 550 (91.7%) smears no such changes were detected. Moreover, in the control group, no atypical epithelial changes were observed. The CCRT-induced oral epithelial atypical changes were predominantly noted at the end of therapy, n = 30 (20%), followed by the 17th day of CCRT, n = 20 (13.3%), in 150 smears on each day (Figure 3).

By applying the Chi-square test for contralateral normal buccal mucosa, a highly significant association was observed between epithelial atypia and radio-chemotherapy dose (p = 0.045), between CCRT-induced epithelial atypical changes and days of treatment (p = 0.001) and between days of CCRT and nuclear atypia (0.000) accordingly. However, no epithelial atypia, nuclear atypia, and epithelial atypical changes were observed in any control group.

## Discussion

Worldwide cancer remains the major reason for death with the sixth most frequent cancer that appears to be the HNC, with an annual incidence of about 630,000 new cases and more than 350,000 deaths [15]. For many years, multiple treatment modalities are used like radiotherapy, chemotherapy, along surgery forming a base for the treatment of OC [16]. Radiotherapy can cause extreme changes in cellular morphology [17]. There are many mucosal abnormalities like epithelial damage as well as inflammation resulting in atypical changes in the areas exposed to radiation [18]. The alterations in the mucosa of the oral cavity may vary as it depends upon multiple factors like types of treatment, radiation, and chemotherapy cycles as well as the concentration of dose [19].

The CCRT-induced molecular and cellular changes include denaturation and coagulation of proteins, nuclear DNA damage, destruction, and inhibition of protein synthesis. Moreover, the normal cells are also affected with subsequent death or they might persist for several years with nuclear and cytoplasmic changes [20].

In the present study, the exfoliative cytology is used on patients who are undergoing CCRT for OSCC because it is a simple and non-invasive method for distinguishing the epithelial cell changes and easily acceptable by the patients [21]. The present study is the first study of its kind carried out in Lahore, Pakistan to investigate the practicality of using oral exfoliative cytology for early detection of oral changes like nuclear atypia, epithelial atypia, and atypical epithelial changes (mild dysplasia) on the contralateral normal buccal mucosa.

The current study reported that the nuclear atypia was raised following the days of CCRT (p = 0.000) which have also been reported in previously conducted studies [19,22]. Radiotherapy induces changes in both cancerous as well as normal cells because of chromosomal injury which can be identified by determining the frequency of micronucleation [16]. The presence of micronucleation is a recognized investigation, carried out for monitoring the efficiency and toxicity of chemopreventive agents as well as the level of genetic alterations caused by radiotherapy [19]. Similar findings were reflected in the current study as the number of micronucleation increases with the increasing dose of CCRT.

The association between epithelial atypia and days of treatment is highly significant for contralateral normal buccal mucosa. In the present study, the epithelial atypia was predominantly observed on the 17th day of CCRT, and this is in accordance with the study conducted by Metgund in India reporting that patients who were exposed to cancer treatments with 28 samples were positive for cytological atypia [19,23]. Likewise, multiple international studies conducted in Sudan, the USA, and Korea suggesting that radiotherapy- and a chemotherapy-induced variable degree of cytological atypia in the normal buccal mucosa, as well as more atypia, was seen in cases treated with combined chemoradiotherapy (p < 0.00001) in contrast to radiation alone [17,22,24-26]. Epithelial atypia is of distinct importance as it may be severe and may be confused with malignancies. The findings of the existing study reveal that patients exposed to chemoradiotherapy might develop oral epithelial atypia as these cancer treatments have the potential to damage not only the rapidly growing cells but also the normal cell population as well and are able for causing tumors.

The condition dysplasia is typically used when the cellular abnormality is limited to the originating tissue. In the present study, atypical epithelial changes were observed mainly at the end of CCRT from the smears taken from the contralateral normal buccal mucosa and these changes might be due to therapy or possibly by contamination of shredded cells from the tumoral area. Very limited data are available on the effect of CCRT-induced atypical epithelial changes in the contralateral normal buccal mucosa. However, the study conducted in Serbia is in conformity with the present study and reported that normal oral mucosa at a distance of 10 mm from the tumor area showed reactive changes, mild dysplasia, and SCC and also reported that smears from the normal-looking oral mucosal tissue taken at different distances from the tumor lesion indicated the existence of subclinical field change [23].

Similar results to the present study were obtained by a study conducted in the UK on patients with unilateral OSCC whose mirror image biopsies were taken from clinically normal-looking mucosa at the corresponding contralateral anatomic site showing histologically abnormal tissue consisting of reactive changes, dysplasia, and early SCC [27]. The studies reported that the cells which are exposed to ionizing radiation show DNA damage, chromosomal aberrations, and increase frequency of mutation and result in the development of sarcoma in the head and neck region [28]. Various studies stated that radiation induces changes in the micro-environment which have an effect on the epithelial cells and cause neoplastic transformation [29].

Slaughter et al. suggested the concept of field cancerization in the OSCC patients and proposed that mucosa of the upper aerodigestive tract has raised the chance of precancerous lesions as numerous genetic alterations occur because of the exposure to carcinogenic agents [30]. Literature research reveals various studies supporting the concept of changes in the field. Thomson while conducting a study on field change and oral carcinogenesis in the UK also revealed that 58% of OC patients have histologically abnormal tissue at the mirror image site [27].

The present study demonstrates that increasing dose of radiotherapy as well as the number of cycles of chemotherapy was associated with various degrees of nuclear atypia, epithelial atypia, and atypical epithelial changes at 17th day and the end of CCRT on the contralateral normal buccal mucosa in contrast to control group. All the previously conducted studies are in accordance with the findings of the present study supporting the concept of field cancerization [23,27].

## Conclusions

In conclusion, it is crucial to be aware of the full spectrum and considerable variability of morphological changes is induced by CCRT. Though CCRT is considered a significant tool for OC management, it may induce sub-clinical field change at distant sites from the tumor lesion. Therefore, serial cytological smears can act as an important diagnostic tool to follow epithelial atypia and atypical epithelial changes in the contralateral normal buccal mucosa of the patients receiving CCRT.
